# Effect of Aromatherapy with Peppermint Oil on the Severity of Nausea and Vomiting in Pregnancy: A Single-blind, Randomized, Placebo-controlled trial

**Published:** 2018

**Authors:** Narges Joulaeerad, Giti Ozgoli, Homa Hajimehdipoor, Erfan Ghasemi, Fatemeh Salehimoghaddam

**Affiliations:** 1- Student Research Committee, Department of Midwifery and Reproductive Health, School of Nursing and Midwifery, Shahid Beheshti University of Medical Sciences, Tehran, Iran; 2- Midwifery and Reproductive Health Research Center, Department of Midwifery and Reproductive Health, School of Nursing and Midwifery, Shahid Beheshti University of Medical Sciences, Tehran, Iran; 3- Traditional Medicine and Materia Medica Research Center and Department of Traditional Pharmacy, School of Traditional Medicine, Shahid Beheshti University of Medical Sciences, Tehran, Iran; 4- Department of Biostatistics, Faculty of Paramedical Sciences, Shahid Beheshti University of Medical Sciences, Tehran, Iran

**Keywords:** Aromatherapy, Inhalation, *Mentha piperita*, Nausea, Pregnancy, Vomiting

## Abstract

**Background:**

Nausea and vomiting are common complaints in the first half of pregnancy. These symptoms can significantly affect a person's personal and professional life. Aromatherapy is one of the types of complementary medicine that is used in the treatment of nausea and vomiting. The objective of this study was to determine the effect of aromatherapy with peppermint oil on the severity of nausea and vomiting of pregnancy (NVP).

**Methods:**

This was a single-blind clinical trial that was conducted on 56 pregnant women with mild to moderate severity of NVP and 6 to 20 weeks of gestational age. After the determination of gestational age and base severity of NVP in each woman, they were randomly assigned to one of the two groups: peppermint oil (n=28) or placebo (n=28). Inhalation aromatherapy was done for four days and at the end of each day, they responded to the Pregnancy Unique Quantification of Emesis/Nausea questionnaire (PUQE). The data obtained were analyzed with Mann-Whitney test and ANOVA with repeated measures using SPSS software version 22. Also, the level of significance was p<0.05.

**Results:**

Although the severity of NVP in each intervention group significantly decreased (p<0.001), the comparison of the severity of NVP during the study period and at the end of it was not statistically significant between the placebo and intervention groups.

**Conclusion:**

According to the possibility of neurological mechanisms causing NVP, the effect of aromatherapy with peppermint oil and placebo were the same in this study. This similarity can be due to psychological impacts of intervention on pregnant women.

## Introduction

Some degree of nausea with or without vomiting occurs in 50 to 90% of all pregnancies and this disorder seems to be more common in western countries and urban areas as compared to African and Asian countries (1). Its most severe form is hyperemesis gravidarum with 1.1% prevalence among people (2). Although the pathogenesis process of NVP is not specified, the main hypotheses on the possible causes of these symptoms include psychological factors, hormonal changes, changes in gastrointestinal motility, and Helicobacter pylori (3). Though NVP is associated with positive implications, these symptoms can significantly affect a person's personal and professional life (4). Studies have shown that nausea and vomiting of pregnancy have a significant impact on family life, the ability to perform usual daily activities, social functioning and stress level. In addition, these symptoms can also affect the quality of life in terms of physiological and mental aspects (5).

Treatment of nausea and vomiting in pregnancy is mainly symptomatic and ranges from dietary changes and oral pharmacological treatment to hospitalization with intravenous fluid replacement and nutrition therapy (6). About half of the drugs used to treat the symptoms of nausea and vomiting are in FDA category C (7). Pregnant women do not want to use drugs during pregnancy, due to the fear of teratogenic effects (8). The use of complementary and alternative medicine is common among women, especially in fertility ages (9). Nowadays around the world, midwives often use complementary therapies in their profession. Aromatherapy is one of the common types of alternative medicines recommended by midwives (10). This method is a branch of herbal medicine which uses the medicinal aspects of essential oils (11).

*Mentha piperita* L. with the common name of peppermint is an aromatic perennial plant with the height of about 1 m that belongs to the Lamiaceae family (12). Herbal therapists use it as antispasmodic, carminative, antiemetic, lactation enhancer, sedative, and for treatment of respiratory and urinary tract infections, morning sickness, dysmenorrhea, diarrhea, and diabetes (13, 14). In the present era, peppermint is considered as a treatment for morning sickness in the British Herbal Pharmacopoeia (15). The plant is classified in B2 Category in terms of its use in pregnancy (16). Peppermint essential oil is obtained from the distillation of aerial parts of the plant (17). The main components of the essential oil are menthol, menthone, and menthyl acetate (18). Inhalation of this essential oil can be used to reduce fever, relieve nausea and vomiting, and improve digestion (12). Peppermint essential oil has antagonistic effects against 5-HT3 receptor channel which can positively affect nausea and vomiting (19). Peppermint essential oil is on the FDA’s generally recognized as safe (GRAS) list (20). The widespread use of complementary medicine methods, especially herbal therapies and insufficient evidence of effectiveness and safety of medicinal herbs during pregnancy are matters which require special attention and more researches seem to be necessary to determine the prevalence, safety, efficacy, and economic benefits of using these methods (21). Given the lack of sufficient studies in this field, the present research was conducted to evaluate the effect of inhalation aromatherapy with peppermint oil on the severity of NVP.

## Methods

This study was a single-blind randomized placebo-controlled clinical trial which was conducted on 56 pregnant women with complaints of NVP, who were referred to selected health centers of Shahid Beheshti University of Medical Sciences in Tehran from December 2014 to the end of May 2015.

Inclusion criteria were age of 18 to 35 years, being Iranian and at least having enough education to read and write, basic level of NVP mild to moderate (score of 3 to 12) based on the score obtained from PUQE questionnaire before the intervention, 6 to 20 weeks of gestational age based on the first day of the last menstrual period (LMP) or ultrasound of the first trimester, having at least one ultrasound report to determine the number and health of fetus, desired pregnancy according to the individual, lack of olfaction problem according to the individual, a normal singleton pregnancy in every respect and no history of obstetric complications in current pregnancy (not having the symptoms of threatened abortion, lack of molar pregnancy), healthy pregnant mother in every respect and no history of known diseases based on information from the records of maternal health profile, no smoking and alcohol consumption, insensitivity to herbal medicine according to the individual, not taking any antiemetic chemical and herbal medicines in 24 *hr* before the start of the study, not having mental health problem and misadventure in six months before the start of the study.

Also, participants were excluded from the study in case of severe nausea and vomiting of pregnancy (a score of 13 or higher on the PUQE questionnaire), sensitivity to essential oil of peppermint or intolerance of peppermint aroma, taking any antiemetic chemical or herbal medicines during the study period, performance of aromatherapy less than three times during the day, occurrence of symptoms of threatened abortion during the study period, and unwillingness to continue to participate in the study.

Tools used in the present study included three questionnaires: (1) Demographic and obstetric questionnaire; (2) PUQE questionnaire; and (3) Final questionnaire. The demographic and obstetric questionnaire consisted of two parts of background information and obstetric history which was completed by the researcher for each woman. The PUQE questionnaire that contains three questions about nausea duration, the number of vomiting and frequency of retching (22, 23). This was used for determining the basic level of NVP and its severity during the study period.

The final questionnaire was used to evaluate the patient satisfaction from treatment, adherence with health and nutritional recommendations, and the evaluation of general changes in symptoms from the viewpoint of pregnant women.

The content validity of the final questionnaire was evaluated. Also, the reliability of the PUQE questionnaire with a correlation coefficient of 0.9 was determined using the test-retest method.

Peppermint essential oil used in the present study was prepared by GiahEsanse Pharmaceutical Company using water distillation from the fresh plant and then, it was diluted with sweet almond oil manufactured by BarijEsanse Pharmaceutical Company with the ratio of 10% and was given to the women in dark amber bottles with an appropriate volume. Also, sweet almond oil was used in the control group as a placebo with the same volume in the same bottle.

The present study was approved by the ethics committee of Shahid Beheshti University of Medical Sciences. After recording the information of the study in the Iranian Registry of Clinical Trials with the number of IRCT201412043860N9, sampling was done with daily referral of researcher to selected health centers using convenience sampling method. Initially, health records of pregnant women were studied and in case of primary qualifications for inclusion, they were asked to answer the PUQE questionnaire to determine the basic severity of NVP. After receiving written consent, the demographic and obstetric questionnaire was completed for each woman and they entered in one of the two intervention or placebo groups using permuted block randomization method. Inhalation aromatherapy was conducted over a period of four days in both groups. In each group, a dark amber bottle with the same volume (filled with peppermint essential oil with a concentration of 10% in the intervention group and sweet almond oil in the placebo group) along with a dropper, a sufficient number of cotton balls and four PUQE questionnaires to determine the severity of NVP were provided for women. They were asked to drop five drops of bottle content on a cotton ball and keep it in one cm under their noses, then have three deep breaths through the nose at the time of nausea feeling four times a day (24). Also, they were asked to complete one of the PUQE questionnaires at the end of each day. Tracking the status of subjects during treatment was done by the researcher using phone. A telephone number was provided to the women in order to contact the researcher if necessary and in case of any questions. Finally, completed PUQE questionnaires were collected and final questionnaires were completed for each woman by the researcher. Descriptive statistics including frequency, mean, standard deviation indexes and statistical analysis tests including independent-samples T-Test, Chi-square, Fisher's exact, Mann-Whitney, and ANOVA with repeated measures were used to analyze the data. Also, before any statistical analysis, the normality of quantitative data was evaluated using Kolmogorov-Smirnov test. SPSS version 22 was used for this purpose and the p<0.05 was considered statistically significant.

## Results

**Figure 1.**
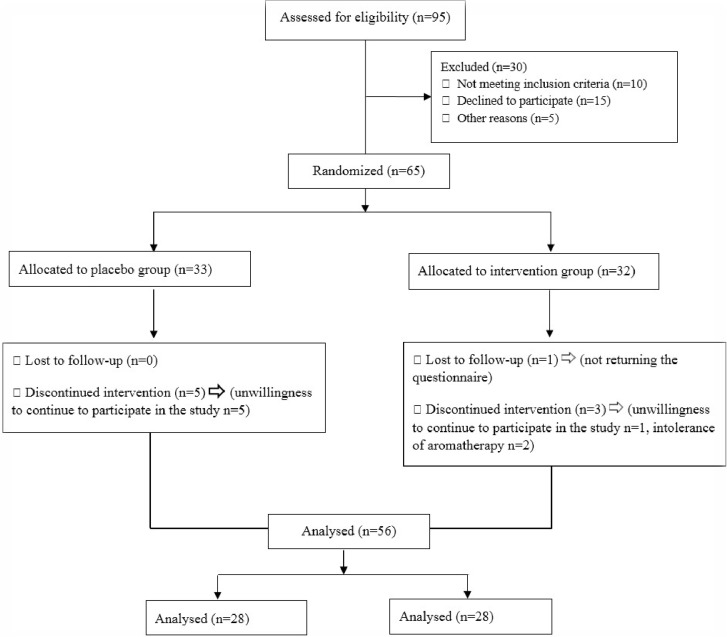
Flow diagram of participants through each stage of randomized controlled trial

The study included 56 participants in two groups of intervention (peppermint essential oil, n=28) and placebo (sweet almond oil, n=28), (Figure 1). Pregnant women in variables such as age, gestational age, education, job status, gravidity, and parity were similar in the pre-intervention stage and there was no significant difference in the two groups in terms of mentioned variables (Table 1).

**Table 1.** Demographic and obstetric characteristics of the two groups (n=56)

**Variable**	**Intervention group**	**Placebo group**	**Test results**
**Age (year)**	26.39±4.27	27.79±3.51	p=0.188
**Education**			p=1.000
Diploma or less	25 (89.3)	26 (92.9)	
University	3 (10.7)	2 (7.1)	
**Job Status**			--
House keeper	28 (100)	28 (100)	
Employed	0	0	
**Gravidity**	1.89±0.68	2.14±0.89	p=0.318
**Parity**	0.82±0.67	0.89±0.73	p=0.734
**Gestational age (week)**	12.4±3.77	12.1±4.06	p=0.658

Data are presented as mean±SD or No. (%)

The average age of the women was 26.39 in the intervention group and 27.79 in the placebo group. Most of them had secondary education level diploma or less in two groups (89.3% and 92.9%, respectively in peppermint oil and placebo groups). All the women in the present study were housewives and most of them were experiencing their second pregnancy. Pregnant women with gestational age of 6 to 20 weeks were examined in this study and permuted block randomization method was used to control this confounding variable.

**Figure 2.**
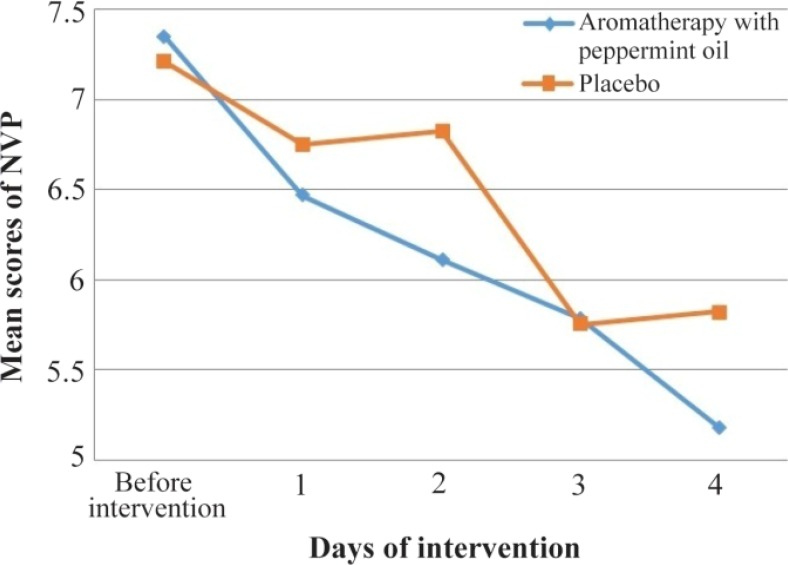
Mean scores of NVP before and during the four-day intervention period in two groups

Mean scores of NVP in peppermint oil group reduced from 7.36 in pre-intervention to 5.18 at the end of four-day intervention period. Also, mean scores at pre-intervention and at the end of the study was 7.21 and 5.82, respectively in the placebo group (Figure 2). ANOVA with repeated measures showed that although the severity of NVP has significantly decreased in each group (p< 0.001), the difference between mean scores of NVP during the four-day intervention period and at the end of it, was not statistically significant between the two groups (p=0.227) (Table 2).

**Table 2.** Comparison of mean difference of scores related to the severity of NVP in two intervention and placebo groups (M±SD)

**Intervention period**	**Intervention group**	**Placebo group**	Test result ^*^
**Before intervention**	7.36±1.92	7.21±1.68	p=1.000 ^b^
**First day**	6.46±2.00	6.75±2.28	
**Second day**	6.11±1.87	6.82±2.35	
**Third day**	5.79±2.00	5.75±2.04	
**Fourth day**	5.18±1.90	5.82±2.14	
Test result ^**^	p<0.001 ^a^	p<0.001 ^a^	p=0.227 ^b^

* Mann-Whitney test,

** ANOVA (Analysis of variance) with repeated measures,

a: Test results in each group,

b: Test results between two groups

Four individuals in the intervention group and one in the placebo group were faced with problems following inhalation aromatherapy. In the intervention group, two individuals complained of headaches, one complained of dizziness on the first day of the intervention and one individual complained of mild shortness of breath immediately after aromatherapy. Also, in the placebo group, one person complained of headache. All items listed were solved in less than 24 *hr* and none of the subjects expressed a desire to leave the study. Data analysis showed that there was no statistically significant difference between the two groups in terms of the mentioned variable (p= 0.352), and this shows the safety of inhalation aromatherapy with peppermint oil in pregnant women. 23 individuals (82.1%) of the pregnant women in peppermint oil group and 24 individuals (85.7%) of the women in the placebo group completely followed health and nutritional recommendations for reducing the severity of NVP.

Generally, 60.7% of pregnant women in peppermint oil group and 57.1% in the placebo group were satisfied with their treatment. So, patient satisfaction from treatment was similar between the two groups.

## Discussion

This research showed that the effect of inhalation aromatherapy with peppermint oil on reducing the severity of NVP is similar to that of placebo.

These results are consistent with an earlier study by Pasha et al. with the difference that researchers used Visual Scale to evaluate the severity of nausea and also counted the number of patients vomiting at various stages of the research (25), while validated PUQE questionnaire was used for this purpose in our research. In this study, pregnant women in the intervention group poured four drops of peppermint oil in a bowl of water and placed it on the ground next to their sleeping place in 4 consecutive nights. Based on the results, the severity of nausea showed a decreasing trend in peppermint and an increasing trend in the control group. The severity of nausea within 7 days after the intervention had a decreasing trend in both groups, but was not statistically significant, so researchers concluded that peppermint oil aromatherapy has not been effective in reducing NVP. Although researchers stated that insufficient sample size is the reason for not achieving the perfect result, it seems that inefficient method for aromatherapy has also been effective in this regard. Considering the fact that symptoms of NVP are not limited to the morning and they mostly occur at all times of the day, in our study, aromatherapy was offered at the time of nausea feeling and pregnant women did not restrict to use it at a specific time.

Ghani and Ibrahim also carried out a similar study on 101 pregnant women (11). In this study, women performed the inhalation aromatherapy for three days with a combination of essential oils of lavender and peppermint. The severity of NVP significantly and considerably decreased on the third day of aromatherapy when compared with the initial data in the intervention group. Perhaps achieving desirable results from this study is because of simultaneous use of lavender and peppermint oils. Also, the method used for aromatherapy in this study was different from ours and oil burner was used for this purpose. Considering this fact that the symptoms of NVP are not limited to a specific time of day and occur in each pregnant women with a different pattern, we used direct inhalation aromatherapy that does not need special equipment, also this method of aromatherapy is accessible and applicable in anytime and anywhere. Another difference is the tool used to evaluate the severity of NVP. We rated the severity of NVP with PUQE questionnaire that is a reliable and standard tool for assessing these symptoms and that was one of the strengths of our study, while Rhodes scale was used for this purpose in this study.

The results also confirm the findings of Anderson and Gross on the effect of aromatherapy on postoperative nausea (26). In this study, 33 subjects were randomly assigned to one of the three groups of aromatherapy with peppermint oil, isopropyl alcohol or saline. The method of aromatherapy was inhalation which is similar to our study and they showed similar effects of aromatherapy in three intervention groups, so researchers concluded that the positive effect of the intervention is mostly related to the breathing patterns, not the specific aroma inhaled.

Also, similar results in the study of Sites et al. have been reported about the controlled breathing with or without peppermint aromatherapy for postoperative nausea (27). In this study, all the subjects received control breathing treatment, but in the intervention group, peppermint spirits aromatherapy was also done. The results showed that the efficacy of breathing technique used in the control group was more than that in the intervention group. Similarly, in our study, the effect of aromatherapy in the intervention and placebo groups on reducing the severity of NVP was similar. Pregnant women participating in our study in both groups had three deep breaths through the nose at the time of aroma inhalation. Thus, achieving the same results in both groups may be related to the effect of the breathing techniques.

Among the limitations in this study, we can mention the difference in psychological status of pregnant women during the study period, as well as the impossibility of blinding for participants due to the nature of direct inhalation aromatherapy. Also, one of the usual limitations of the studies in the field of aromatherapy is the individual's positive or negative attitude toward a special aroma which varies from one person to another due to different preferences.

Considering the reducing trend of the severity of NVP during the present study, more precise findings can be achieved by further trials with longer intervention period and larger sample size. Also, further investigations on different pharmaceutical forms of peppermint or other kinds of aromatherapy with peppermint oil would contribute to establishing more accurate results in this issue.

## Conclusion

The findings suggest that despite a significant decrease in the severity of NVP in each intervention group, this decreasing trend between the two groups was not statistically significant and the effect of inhalation aromatherapy with peppermint oil on reducing the severity of nausea and vomiting of pregnancy was similar to that of placebo. It seems that this similarity was found due to the possibility of neurological mechanisms causing nausea and vomiting in pregnancy. Therefore, the empowerment of women to maintain mental relaxation during pregnancy is recommended so that the severity of NVP may be reduced. For this purpose, breathing methods and relaxation techniques can be considered in prenatal training programs.
